# Risk of Heart Failure Hospitalization Associated With Cilostazol in Diabetes: A Nationwide Case–Crossover Study

**DOI:** 10.3389/fphar.2018.01467

**Published:** 2019-01-07

**Authors:** Cho-Kai Wu, Jou-Wei Lin, Li-Chiu Wu, Chia-Hsuin Chang

**Affiliations:** ^1^Department of Internal Medicine, National Taiwan University Hospital, Taipei, Taiwan; ^2^Department of Medicine, College of Medicine, National Taiwan University, Taipei, Taiwan; ^3^Cardiovascular Center, National Taiwan University Hospital Yunlin Branch, Douliu, Taiwan; ^4^Institute of Epidemiology and Preventive Medicine, College of Public Health, National Taiwan University, Taipei, Taiwan

**Keywords:** heart failure hospitalization, cilostazol, diabetes, case-crossover study, outcomes

## Abstract

**Background and Objective:** It has been suggested to avoid cilostazol, the first-line therapy for peripheral arterial disease, in patients with congestive heart failure (HF). The objective of this study was to evaluate the risk of hospitalization for heart failure (HHF) associated with cilostazol use in the patients of diabetes mellitus.

**Methods:** This case-crossover study retrieved records on diabetic patients > 20 years of age who were hospitalized for heart failure during the period of 2009–2011 from the Taiwan National Health Insurance Database. The “current” period was defined as 1–30 days prior to HHF whereas the 91–120 days prior to HHF served as the “reference” period. The exposure status just preceding the event is compared with exposure of the same person in one or more referent remote to the event. Adjusted odds ratios (OR) were used to estimate time-varying discordant exposure by the ratio of the number exposed to cilostazol only during the case period to the number exposed to cilostazol only during the control period.

**Results:** A total of 47,506 diabetic patients were included in the analysis (average age: 72.7 ± 12.4, percentage of males: 48%). A total of 399 patients (0.84%) received cilostazol only in the current period, and 252 (0.53%) received cilostazol only in the reference period. After adjustment for other medications, a significant association was found between cilostazol and HHF (OR: 1.35, 95% CI: 1.14–1.59). After further adjustment for time-varying co-morbidities the ORs remained essentially the same. Sensitivity analyses using different definitions of control period (ranging from 31–60, 61–90, to 121–150 days before index date) yielded adjusted ORs of 1.43 (95% CI: 1.14–1.79), 1.31 (95% CI: 1.09–1.57) and 1.23 (95% CI: 1.06–1.44), respectively suggesting the robustness of our study findings.

**Conclusion:** Use of cilostazol may be positively related to the risk of HHF. Further studies are warranted to explore the underlying mechanisms and to confirm the association.

## Introduction

Cilostazol is a unique antiplatelet agent that has been commercially available for two decades. It is a potent antiplatelet agent and possesses vasodilatation and antiproliferative effects. It has been broadly applied in the treatment of peripheral artery disease (PAD) and also for patients with PAD undergoing endovascular procedures. In addition, a randomized placebo controlled trial has investigated the role of cilostazol in patients with coronary artery disease (CAD) undergoing primary coronary intervention (PCI) along with the use of aspirin and/or clopidogrel (Rogers et al., 2015).

Cilostazol has multifactorial pharmacological properties and broad spectrum of pharmacological actions. Its main effect includes selective inhibition of cellular phosphodiesterase (PDE) type III, which augments the effect of cyclic adenosine monophosphate (cAMP) metabolism within the platelet (Pan et al., 1994; Schror, 2002). Since cAMP is involved in the pathway which controls platelet aggregation and vasomotor function, inhibition of PDE III could reversibly inhibit platelet aggregation and results in vasodilation (Schror, 2002). Besides, cilostazol has also been shown to reduce triglycerides, raise high-density lipoprotein levels and increase adenosine accumulation in the interstitium, all of which provide protective effects for patients with stroke, CAD and PAD. Nevertheless, inhibition of PDE III may lead to a positive inotropic effect, meta-analysis for randomized trials of PDE III inhibitors versus placebo in heart failure (HF) patients revealed that PDE III inhibitors were responsible for an increase in mortality rate compared with placebo in patients suffering from chronic HF. Thus, the use of PDE III inhibitors is prohibited in HF patients. The above results were mainly obtained from the trials involving intravenous medication for HF (e.g., milrinone, vesnarinone). Whether the use of cilostazol is associated with development of HF or worsening of HF is still unknown.

Diabetes is a major risk factor for development of PAD. The number of prescriptions for cilostazol in diabetic patients have increased gradually and, therefore, understanding the role of cilostazol in diabetic patients has become even more important. Therefore, this national-wide population-based study aimed to examine whether the use of cilostazol was associated with hospitalization for heart failure (HHF) in the diabetic population.

## Patients and Methods

This study was approved by our hospital’s Research Ethics Committee. The case-crossover study design was used to assess the relationship between cilostazol exposure and risk of HF that led to hospitalization. Instead of using propensity-matched controls, each patient served as his/her own control in the case-crossover study, so that stable confounders, unmeasured or poorly measured, would not influence the results. In addition, because diabetic patients might receive complicated anti-hypertensive and anti-diabetic drug therapy and switch from one class of drug to other classes in order to attain optimal blood pressure and glycemic control, it was difficult to find appropriate comparison groups. The major advantage of this design is that stable risk factors such as cigarette smoking or other cardiovascular risk factors will not confound the analysis results, despite the fact that they are not recorded in the claim database.

### Data Source

A universal NHI program was implemented in Taiwan in March 1995. Ninety-six percent of the total Taiwanese population was enrolled in this NHI program. The Taiwan NHI database includes complete outpatient visits, hospitalization data, prescriptions, and disease status for 99% of the 23 million inhabitants of Taiwan. The longitudinal medical history of each beneficiary was established by linking several computerized administrative, claims datasets and National Death Registry through the civil identification number unique to each beneficiary and their date of birth. Data for gender, birth date, medications, and diagnostic codes (based on the International Classification of Diseases, Ninth Revision, Clinical Modification) were retrieved for the analyses performed in this study. Patients were required to have at least 1 year of registration in the National Health Insurance database prior to index date.

### Study Population

**FIGURE 1:**
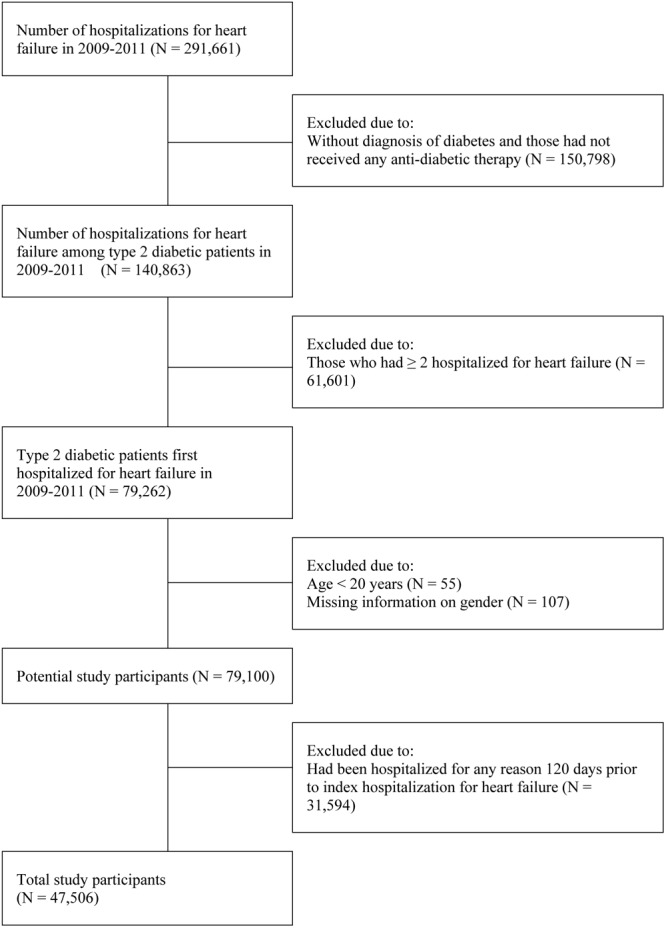
Study flow.

As shown in Figure 1, all patients hospitalized for CHF in 2009–2011 were included in the study, based on having an International Classification of Diseases, Ninth Revision, Clinical Modification (ICD-9-CM) code of 428 on inpatient claims. Previous validation studies using a hospital administrative database reported a positive predictive value of 90% with this definition (Wu et al., 2015). For those who had ≥2 hospitalizations for heart failure during the study period, only the first event was included. Date of hospitalization was defined as the index date. Patients who did not have ICD-9-CM code of diabetes (250) and those had not received any anti-diabetic therapy before hospitalization, patients aged < 20 years, and those with missing information regarding gender were excluded. Patients were also excluded: (1) if they were admitted for any reason during 120 days before index date due to clinical instability or (2) due to the inability to ascertain dosage and duration of cilostazol use during hospitalization. We also repeat the same analysis and included patients with mortality and those without to test the effect of cilostazol.

### Data on Drug Exposure and Confounding Factors

The main exposure of interest in this study was cilostazol use (reimbursed by NHI since 2001 for the indication of relieving intermittent claudication symptoms). Because non-steroidal anti-inflammatory drugs (NSAIDs) have had documented association with CHF, the risk estimates for NSAIDs were calculated as a comparison. Information regarding the type of drug prescribed (according to the anatomic therapeutic chemical ATC classification system), date of prescription, days of supply, and total number of drug pills dispensed from pharmacy prescription database were collected. Other concomitant drug exposure that may also modify the risk of HF are listed in Table 2 and include insulin, oral antidiabetic drugs, antiplatelet agents (aspirin, clopidogrel), warfarin, anti-hypertensives, nitrates, lipid-lowering agents, digitalis, anti-arrhythmics, inhaled and oral bronchodilators (beta-adrenergic agonists, inhaled anticholinergics, aminophylline), oral corticosteroids, and systemic antibiotics (ATC codes were provided in Supplementary Table 1).

McNemar’s tests were used for comparison of drug exposure between case and control period. Information on patient’s age, gender, and comorbidities (including hypertension, ischemic heart disease, myocardial infarction, atrial fibrillation, congestive HF, cerebrovascular disease, ischemic stroke, intracerebral hemorrhage, peripheral vascular disease, chronic renal, liver, and lung disease, depression, and cancer based on ICD-9-CM codes) were also collected (Supplementary Table 1) (Wu et al., 2010). The Charlson’s index (ranging from 0 to 40) was used as a measure of co-morbid conditions, such as chronic liver disease, stroke, or cancer (a total of 22 conditions). Each condition was assigned a score of 1, 2, 3, or 6, depending on the mortality associated with each one. Scores were summed to provide a total score that predicted long-term mortality (Chang et al., 2010).

### Statistical Analysis

In the case-crossover design, the exposure status just preceding the event (defined as “case period”) is compared with exposure of the same person in one or more referent “control periods” remote to the event. The odds ratios (ORs) and their 95% confidence interval (CIs) were estimated by the ratio of the number exposed to cilostazol only during the case period to the number exposed to cilostazol only during the control period (i.e., ratio of discordant pairs) by conditional logistic regression. In the main analysis, case period was defined as 1–30 days prior to index date and control period was defined as 91–120 days prior to index date. Persons were considered currently exposed to cilostazol and other concomitant drugs during the start date and end date of a prescription.

In the multivariable analysis, adjusted ORs were calculated simultaneously and controlled for time-varying discordant exposure to medications including metformin, pioglitazone, sitagliptin, aspirin, angiotensin-converting enzyme inhibitors, beta-adrenergic blockers, calcium channel blockers, diuretics, nitrates, digitalis glycoside, inhaled and oral bronchodilators (beta-adrenergic agonists, anticholinergics, aminophylline), oral corticosteroids, and systemic antibiotics between case and control periods except for the medication being analyzed itself. Further adjustment for time-varying confounding co-morbidities (hypertension, ischemic heart disease, cerebrovascular disease, chronic kidney disease) or adjustment for surrogate markers of glycemic control (number of outpatients visits and the number of A1c tests prescribed) were also performed.

In sensitivity analyses, different control periods of 31–60, 61–90, and 121–150 days prior to index date were used, and recent 7 days of drug exposure were excluded to avoid protopathic bias (physicians discontinued cilostazol therapy because of early symptoms/signs of HF) to see whether results would change substantially. In another sensitivity analyses, longer case and control period windows (i.e., 60 or 90 days) were used to capture the risk associated with longer-term exposure of cilostazol (Chang et al., 2015).

Furthermore, stratified analysis was performed to evaluate potential modification effects. The cases were separated according to (1) gender (men vs. women), (2) age (≥65, <65 years), (3) congestive HF, and (4) chronic kidney disease. A formal test of interaction was performed for each subgroup to examine if the difference in size of effect between two subgroups was statistically significant. A two-sided *p*-value < 0.05 was considered to be statistically significant. All statistical analyses were performed with SAS 9.2 (SAS Institute, Cary, NC, United States).

## Results

A total of 79,100 patients with diabetes who were ≥ 20 years of age and who were first hospitalized for CHF between 2009 and 2011 were identified. After further excluding those who had been hospitalized for any reason 120 days prior to index HHF, a total of 47,506 patients (48.2% male, mean age 72.7 years) were included in the final analysis (Figure 1).

Table 1 summarizes the proportion of patients with comorbidities at index hospitalization. Approximately 63.7% of the cohort had hypertension, 47.3% had ischemic heart disease, 12.4% had a history of myocardial infarction, and 20.5% had chronic kidney disease. The exposure of cilostazol, anti-hypertensive, and anti-diabetic agents, as well as concomitant medications during the case period (1–30 days before index hospitalization) and control period (91–120 days before index hospitalization) is shown in Table 2. A higher proportion of patients took cilostazol, anti-diabetic agents (except for metformin), aspirin, angiotensin converting enzyme inhibitors, beta-blockers, calcium channel blockers, diuretics, nitrates, digitalis, inhaled bronchodilators, aminophylline, systemic antibiotics, and NSAIDs in the case period than in the control period.

**Table 1:** Characteristics of the diabetic patients hospitalized for congestive heart failure (*N* = 47,506).

Characteristics	%
Age (mean ± SD)	72.70 ± 12.41
Male (%)	48.23
Comorbidity (%)	
Atrial fibrillation	14.19
Cancer	5.25
Chronic kidney disease	20.54
Chronic liver disease	4.07
Chronic lung disease	20.26
Depression	2.19
Hypertension	63.74
Intracerebral hemorrhage	1.06
Ischemic heart disease	47.32
Ischemic stroke	9.34
Myocardial infarction	12.35
Peripheral vascular disease	1.23
Charlson’s index (mean ± SD)	3.27 ± 1.58
Number of different ICD-9 diagnoses (mean ± SD)	8.10 ± 2.45
Number of cardiovascular-related diagnoses (mean ± SD)	2.91 ± 1.22

**Table 2:** Proportion of patients with concomitant medications use and resource utilization during 1–30 and 91–120 days before hospitalization for congestive heart failure (*N* = 47,506).

	Case period	Control period
Concomitant medication use	(1–30 days before	(91–120 days before
and resource utilization	index day)	index day)
**Concomitant medications use (%)**		
Aspirin	37.59	33.99
Clopidogrel	8.37	7.82
Cilostazol	3.96	3.65
Warfarin	4.90	4.68
Angiotensin converting enzyme inhibitors	14.05	12.94
Angiotensin receptor blockers	6.86	6.37
Alpha-blockers	7.40	6.81
Beta-blockers	37.45	33.02
Calcium channel blockers	44.75	42.71
Diuretics	48.19	40.06
Other anti-hypertensive agents	3.17	2.57
Metformin	30.82	30.91
Sulfonylurea	36.39	36.10
Alpha-glucosidase inhibitors	10.52	10.11
Pioglitazone	5.98	5.74
Glinides	8.79	8.47
Sitagliptin	6.05	4.88
Insulin	16.61	15.23
Nitrates	29.59	24.02
Statins	22.99	22.25
Fibrates	5.24	5.31
Digitalis glycoside	12.12	10.55
Antiarrhythmics class I and III	5.85	4.77
Inhaled beta-agonists	6.98	3.85
Inhaled anticholinergics	2.85	1.13
Inhaled corticosteroids	0.46	0.33
Aminophylline	18.65	12.30
Oral corticosteroids	9.42	6.92
Systemic antibiotics	19.70	11.75
Non-steroid antiinflammatory drugs	30.37	22.57
**Resource utilization**		
Mean number of outpatient visits	5.73	4.59
Mean number of A1c tests	0.25	0.22
Number of outpatient visits ≥ 2 (%)	9.47	3.61
Number of A1c tests ≥ 1	13.39	11.21

Table 3 presents crude and adjusted ORs and their 95% CIs for cilostazol and NSAIDs by conditional logistic regression. A modestly increased risk of hospitalization for HHF was found for the exposure of cilostazol with a crude OR of 1.57 (95% CI: 1.35–1.84). After controlling for potential time-varying confounders including drugs potentially associated with HF, cilostazol was associated with an increased risk of HHF (OR: 1.35, 95% CI: 1.14–1.59) for hospitalized CHF. A significantly elevated OR was also evident for NSAIDs (adjusted OR 1.69, 95% CI: 1.62–1.76).

**Table 3:** Risk of hospitalized heart failure associated with current use of cilostazol and NSAIDs (*N* = 47,506).

	Number of patients exposed to the medication			Crude odds ratio		Adjusted odds ratio^∗^	
	During case (1–30) period or control period (91–120) (%)	During case (1–30) period but not control period (91–120) (%)	During control period (91–120) but not case period (1–30) (%)	Point estimate	95% C.I.	Point estimate	95% C.I.
Cilostazol	4.50	0.84	0.53	**1.57**	1.35–1.84	**1.35**	1.14–1.59
NSAIDs	38.69	16.11	8.32	**1.94**	1.86–2.01	**1.69**	1.62–1.76

*^∗^Conditional logistic regression adjusted for important potential time-varying confounding variables including pioglitazone, metformin, sitagliptin, aspirin, angiotensin converting enzyme inhibitors, beta-blockers, calcium channel blockers, diuretics, nitrates, digitalis glycoside, inhaled beta-agonists, inhaled anticholinergics, aminophylline, oral corticosteroids, and systemic antibiotics. Bold values indicate statistical significance; OR, odds ratio, 95% C.I., confidence interval.*

After further adjustment for time-varying co-morbidities including hypertension, ischemic heart disease, cerebrovascular disease, chronic kidney disease, or further adjustment for surrogate measures of glycemic control including number of outpatients visits and the number of A1c test prescribed, the ORs remained essentially the same (Supplementary Table 2).

Sensitivity analyses using different definitions of control period (ranging from 31–60, 61–90, to 121–150 days before index date) yielded adjusted ORs of 1.43 (95% CI: 1.14–1.79), 1.31 (95% CI: 1.09–1.57) and 1.23 (95% CI: 1.06–1.44), respectively (Table 4A). An analysis excluding recent 7 days of drug exposure prior to index date showed similar results suggesting the robustness of our study findings (Table 4A). Additional sensitivity analyses using longer case and control time windows (i.e., 60 and 90 days) showed a similar risk estimate in the analysis using a window length of 60 days; however, the OR were attenuated and became non-significant in the analysis using a window length of 90 days (Table 4B). In contrast, NSAIDs were consistently associated with significantly higher risks, regardless of the definition and window length used for the case and control periods.

**Table 4A:** Risks of hospitalized heart failure associated with cilostazol and NSAIDs use on different definitions of case period and control period (*N* = 47,506).

	Case period 1–30 days		Case period 1–30 days		Case period 1–30 days		Case period 8–30 days	
	before index date		before index date		before index date		before index date	
	Control period 31-60 days		Control period 61-90 days		Control period 121-150 days		Control period 98-120 days	
	before index date		before index date		before index date		before index date	
	Adjusted^∗^ OR	95% C.I.	Adjusted^∗^ OR	95% C.I.	Adjusted^∗^ OR	95% C.I.	Adjusted^∗^ OR	95% C.I.
Cilostazol	**1.43**	1.14–1.79	**1.31**	1.09–1.57	**1.23**	1.06–1.44	**1.27**	1.08–1.50
NSAIDs	**1.70**	1.62–1.78	**1.67**	1.60–1.74	**1.62**	1.56–1.68	**1.34**	1.29–1.40

*^∗^Conditional logistic regression adjusted for important potential time-varying confounding variables including pioglitazone, metformin, sitagliptin, aspirin, angiotensin converting enzyme inhibitors, beta-blockers, calcium channel blockers, diuretics, nitrates, digitalis glycoside, inhaled beta-agonists, inhaled anticholinergics, aminophylline, oral corticosteroids, and systemic antibiotics. Bold values indicate statistical significance; OR, odds ratio; 95% C.I., confidence interval.*

**Table 4B:** Risks of hospitalized heart failure associated with cilostazol and NSAIDs use on different window lengths of case period and control period (*N* = 47,506).

	Case period 1-60 days		Case period 1-60 days		Case period 1-90 days		Case period 1-90 days	
	before index date		before index date		before index date		before index date	
	Control period 61-120 days		Control period 91-150 days		Control period 91-180 days		Control period 121-210 days	
	before index date		before index date		before index date		before index date	
	Adjusted^∗^ OR	95% C.I.	Adjusted^∗^ OR	95% C.I.	Adjusted^∗^ OR	95% C.I.	Adjusted^∗^ OR	95% C.I.
Cilostazol	**1.54**	1.27–1.88	**1.31**	1.12–1.54	1.09	0.93–1.27	1.06	0.92–1.21
NSAIDs	**1.51**	1.45–1.58	**1.45**	1.39–1.50	**1.33**	1.28–1.38	**1.26**	1.22–1.31

*^∗^Conditional logistic regression adjusted for important potential time-varying confounding variables including pioglitazone, metformin, sitagliptin, aspirin, angiotensin converting enzyme inhibitors, beta-blockers, calcium channel blockers, diuretics, nitrates, digitalis glycoside, inhaled beta-agonists, inhaled anticholinergics, aminophylline, oral corticosteroids, and systemic antibiotics. Bold values indicate statistical significance; OR, odds ratio; 95% C.I., confidence interval.*

The subgroup analysis showed that the increased risk of HHF associated with cilostazol exposure was uniform across pre-defined strata by gender, age, prior history of HF, and chronic kidney disease, suggesting no significant modification effect by these characteristics (Table 5). The same analysis was performed to analysis cilostazol’s effect for overall mortality (Supplementary Table 3). Similar findings were found and recent us of cilostazol was associated with an OR of 3.90 (95% CI: 3.51–4.33) for increased mortality risk (Supplementary Table 4). The results were consistent after adjustment for comorbidities and drug use (Supplementary Table 5).

**Table 5:** Adjusted odds ratio of hospitalized heart failure associated with use of cilostazol and NSAIDs among different subgroups.

	Men (*N* = 22,911)		Women (*N* = 24,595)		*P*-values for interaction
	Adjusted OR	95% C.I.	Adjusted OR	95% C.I.	
Cilostazol	**1.43**	1.11–1.84	**1.29**	1.03–1.63	0.56
NSAIDs	**1.67**	1.57–1.77	**1.70**	1.61–1.80	0.65

	**Age ≥ 65 (*N* = 35,718)**		**Age < 65 (*N* = 11,788)**		
Cilostazol	**1.37**	1.13–1.65	1.27	0.88–1.85	0.72
NSAIDs	**1.68**	1.60–1.76	**1.69**	1.56–1.85	0.69

	**Patients with heart failure (*N* = 9,164)**		**Patients without heart failure (*N* = 38,342)**		
Cilostazol	**1.65**	1.10–2.47	**1.29**	1.07–1.56	0.28
NSAIDs	**1.66**	1.51–1.84	**1.70**	1.62–1.78	0.61

	**Patients with chronic kidney disease (*N* = 9,757)**		**Patients without chronic kidney disease (*N* = 37,749)**		
Cilostazol	**1.52**	1.14–2.04	**1.27**	1.03–1.56	0.33
NSAIDs	**1.55**	1.41–1.70	**1.72**	1.65–1.81	0.05

*Conditional logistic regression adjusted for important potential time-varying confounding variables including pioglitazone, metformin, sitagliptin, aspirin, angiotensin converting enzyme inhibitors, beta-blockers, calcium channel blockers, diuretics, nitrates, digitalis glycoside, inhaled beta-agonists, inhaled anticholinergics, aminophylline, oral corticosteroids, and systemic antibiotics. Bold values indicate statistical significance; OR, odds ratio; 95% C.I., confidence interval.*

## Discussion

In this study, we analyzed a nationwide health insurance claims database and found an increased risk of HHF with use of cilostazol in diabetic patients. The effect of cilostazol was only slightly lower than that associated with the use of non-steroid anti-inflammatory drugs (NSAID). We also observed that the elevated risk of HHF associated with cilostazol exposure was uniform across all pre-defined subgroups after controlling for potential risk factors.

PDE3-selective inhibitors such as milrinone and vesnarinone have been used clinically to treat acute congestive HF (Benotti et al., 1978). Blockage of PDE 3 could lead to increased levels of intracellular cAMP and protein kinase A which could result in smooth muscle cell relaxation and attenuation of myosin phosphorylation (McDaniel et al., 1994). On the other hand, elevated cAMP levels enables L-type Ca2+ channels and a component of a delayed-rectifier potassium channel signal transduction pathway in the sinoatrial node (Kodama-Takahashi et al., 2003). Therefore, drugs that inhibit PDE 3 pathways would cause a positive chronotropic effect and inotropic action which have been demonstrated to increase left ventricular ejection fraction and stroke volume (Baim et al., 1983; Jaski et al., 1985; Rauch et al., 1991). However, clinical studies [such as the Prospective Randomized Milrinone Survival Evaluation (PROMISE) (Packer et al., 1991) and the Vesnarinone trial (VEST) (Feldman et al., 1993) studies] have evaluated the long-term effects of PDE 3 administration in congestive HF patients and reported increased cardiovascular mortality. Several studies have also shown that long-term elevation of cAMP could cause adverse effects on chronic HF patients (Movsesian, 2003; Phrommintikul and Chattipakorn, 2006). Increased levels of cAMP trigger early and delayed after-depolarization and enhance the risk of lethal ventricular arrhythmia. Besides, the inotropic effects of PDE 3 inhibitors could cause higher myocardial oxygen consumption and increase various arrhythmia over time (Naccarelli and Goldstein, 1989; Sunderdiek et al., 2000).

Cilostazol also inhibits PDE 3 selectively. It has potent vasodilating and antithrombotic effects and has been used in the treatment of PAD (Dawson et al., 1998). Therefore, the FDA contraindicated the use of cilostazol in HF of any severity. Nevertheless, no clinical study has directly investigated the effects of cilostazol in HF patients. Although cilostazol is a PDE 3 inhibitor, the effect on myocytes differs from other PDE 3 inhibitors. The weaker inotropic effect of cilostazol compared with milrinone lessens long-term cardiac side effects (Cone et al., 1999). Cilostazol also blocks adenosine uptake, and, subsequently, the interstitial adenosine elevation in the heart could attenuate cAMP response and limit the long-term harmful effects of cAMP activation (Wang et al., 2001). In addition, in smooth muscle and platelets, elevated interstitial and circulating adenosine levels by cilostazol could potentiate its cAMP-raising effect through inhibiting PDE3 and, thereby, augmenting antiplatelet and vasodilatory effects which could be beneficial for prognosis of congestive HF (Liu et al., 2001). For subjects with myocardium ischemic/reperfusion, cilostazol could decreased plasma IL-6, IL-1beta and TNF-alpha levels, and activate the PPARγ/JAK2/STAT3 pathway. Through the above pathway, cilostazol is possible to limit myocardial inflammation and apoptosis after ischemic/reperfusion injury. These results may provide a rationale for new therapy with cilostazol in patients with ischemic cardiomyopathy and may possible restore cardiac function (Li et al., 2017). Taken together, the use of cilostazol in distinct groups should be reinvestigated.

The biological mechanism(s) behind the increased risk of HHF associated with cilostazol is unclear. Atarashi et al. (1998) demonstrated that cilostazol increased heart rate and improved symptoms in patients with symptomatic bradyarrhythmia. There is no cardiac electrophysiological study that has investigated the strength of cilostazol. In contrast, there is evidence for its possible undesirable tachyarrhythmia effects (Barta et al., 2008; Shinohara et al., 2010). Elevated resting heart rate was associated with increased risk for incident HF in asymptomatic high risk patients and higher heart rate was an independent risk factor for HHF in the subclinical group (Opdahl et al., 2014). Few studies specifically evaluated the effect of cilostazol on HF in the patients with diabetes. One recent meta-analysis evaluated 10 randomized case-control trials in the diabetic population, mostly with reported outcomes of major adverse cardiovascular events or platelet aggregation function (Bundhun et al., 2015). However, all the above trials failed to provide information about the risk of HHF associated with cilostazol use.

Our current study compared recent use of cilostazol (1–30 days) with controls (remote use) to document the influence of the drug over a short period. In the sensitivity analysis, we excluded the recent 7 days of drug exposure and found similar risk for the drug. We also used different definitions of control periods or longer case and control time windows (i.e., 60 and 90 days), assuming the statistical significance associated with discordant cilostazol use might diminish. In contrast, the effects of NSAIDs were consistently associated with significantly higher risks, regardless of the definition or window length used for the case and control periods. These findings suggested that exposure to cilostazol caused an accumulated HHF risk with a duration about 2 months but not longer. Instead, the well-known risks for NSAID in diabetic patients are consistent around 3 months. As we have discussed, cilostazol has a much weaker inotropic effect than other PDE3 inhibitors and could lessens long-term cardiac side effects. However, cilostazol might also increase heart rate and hence lead to higher HHF risk during the initial 2-month period. On the other hand, our data was consistent with the previous conclusion that NSAID use is associated with a higher risk of myocardial infarction, stroke, and hospitalizations for both ischemia and HF (Kohli et al., 2014) which further validated the reliability of our cohort study.

A very recent study used five population-based healthcare databases from four European countries and showed that current use of any NSAID (use in preceding 14 days) was found to be associated with a 19% increased risk of HHF (adjusted OR: 1.19, 95% CI: 1.17–1.22), compared with past use of any NSAIDs (use > 183 days in the past). The results were consistent with our findings of an association between NSAID use and HHF. Hence, we verified the higher risk of developing HHF associated with NSAID use compared with that of cilostazol in diabetic patients (Arfe et al., 2016).

### Limitation

Our study had several limitations. First, we did not have information on lifestyle risk factors such as weight, cigarette smoking, and alcohol consumption. However, these lifestyle factors would probably not change substantially during the relatively short study period and thus could be controlled by our case-crossover design. We also relied on the classification from the claims data base for baseline characteristics which may have resulted in a potential bias in terms of disease classification. Usually, doctors will exclude reversible causes for diagnosis of HHF. However, because detailed hematology data were not available, it was not possible to adjust for some potential confounding factors of HHF or mortality. Second, our case-control design evaluated the effect of cilostazol over a certain period. This method justified the effect of cilostazol as compared with controls, but the long-term risk concerning cilostazol is still unknown. In addition, from the results of current study, we suggested that recent use cilostazaol seems to be one of the determinants of HHF. However, because no data on inflammation parameters were available, all proposed mechanisms are still hypothetical. Although we used a cross-over design to control for the most important risk factors for HHF such as age, HTN and comorbid conditions, some unknown factors that may influence the outcomes in this population such as obesity, smoking, alcohol consumption, family history of premature CAD, lifestyle and diet, might still affect the results of current study. Finally, we could not exclude possible time-varying within-subject confounding factors that were associated with disease severity over time or trends in cilostazol use. The multivariable analysis might also over-control some of the intermediate variables responsible for HHF associated with cilostazol.

### Strength

One of the strength of present study is that we performed the largest National-wide case-crossover study that included 47,506 diabetic patients to find significant association between cilostazol and HF hospitalization. We showed for the first time that use of cilostazol may be positively related to the risk of heart failure hospitalization.

## Conclusion

In conclusion, our study found that cilostazol use was associated with a modestly increased risk of HHF. Further studies are warranted to explore underlying mechanisms and to confirm the association.

## Author Contributions

C-HC had full access to all of the data in the study and takes responsibility for the integrity of the data and the accuracy of data analysis. C-KW and J-WL participated in the study concept and design. L-CW acquired the data. C-KW and J-WL also contributed to the analysis and interpretation of data.

## Conflict of Interest Statement

The authors declare that the research was conducted in the absence of any commercial or financial relationships that could be construed as a potential conflict of interest.
