# Supplementary material for: Risk of Heart Failure Hospitalization Associated With Cilostazol in Diabetes: A Nationwide Case–Crossover Study
Source: Front Pharmacol. 2019 Jan 7;9:1467. doi: 10.3389/fphar.2018.01467 (PMC6330376; doi:10.3389/fphar.2018.01467)
Supplement: Supplementary file 1 [file Table_1.DOCX]

**Supplementary Table 1.** ICD-9-CM codes and ATC codes used in this study

| **Comorbidities** | **ICD-9-CM codes** | **Medication** | **ATC codes** |
| --- | --- | --- | --- |
| Diabetes | 250 | Angiotensin receptor blockers | C09CA01, C09CA03, C09CA04, C09CA06, C09CA07, C09CA08, |
| Hypertension | 401-404 | ACE inhibitors | C09AA |
| Ischemic heart disease | 410-414 | Alpha-blockers | C02CA |
| Myocardial infarction | 410, 412 | Beta-blockers | C07A |
| Congestive heart failure | 428 | Calcium channel blockers | C08 |
| Atrial fibrillation | 427.31 | Diuretics | C03 |
| Cerebrovascular disease | 430-438 | Other anti-hypertensive agents | C02A, C02B, C02CC, C02D, |
| Ischemic stroke | 433, 434, 436 (exclude 800, 801, 802, 803, 804, 850, 851, 852, 853, 854 ,V57) | Nitrate | C01DA |
| Intracerebral hemorrhage | 430-432 | Insulin | A10A |
| Peripheral arterial disease | 440.2, 440.4, 443.81, 443.9 | Sulfonylurea | A10BB |
| Chronic renal failure | 403.01, 403.11, 403.91, 404.02, 404.03, 404.12, 404.13, 404.92, 404.93, 585 ,V45.1, V56.0, V56.8 | Metformin | A10BA02 |
| Chronic liver disease | 070.2x, 070.3x, V02.61, 070.41, 070.44, 070.51, 070.54, V02.62, 571.0, 571.1, 571.2, 571.3, 571.4, 571.5, 571.6 | Thiazolidinediones | A10BG02, A10BG03 |
| Chronic lung disease | 490-496, 500-508 | Glinides | A10BX02, A10BX03 |
| Depression | 296.2,296.3,298.0,300.4,309.0,309.1, 293.83,296.90,309.28,296.82, 311 | Alpha-glucosidase inhibitors | A10BF |
| Cancer | 140-208 | Sitagliptin | A10BH01 |
|  |  | Statins | C10AA |
|  |  | Fibrates | C10AB |
|  |  | Warfarin | B01AA03 |
|  |  | Aspirin | B01AC06, N02BA01 |
|  |  | Clopidogrel | B01AC04 |
|  |  | Cilostazol | B01AC23 |
|  |  | COX-2 selective and nonselective NSAIDs | M01A (exclude M01AX05) |
|  |  | Digitalis | C01AA |
|  |  | Anti-arrhythmics Class I and III | C01B |

**Supplementary Table 2.** Adjusted odds ratio of hospitalized heart failure associated with current use of cilostazol and NSAIDs (N = 47,506)

|  | Adjusted OR^a^ | | Adjusted OR^b^ | |
| --- | --- | --- | --- | --- |
|  | Point estimate | 95% C.I. | Point estimate | 95 % C.I. |
| Cilostazol | **1.35** | 1.14-1.59 | **1.35** | 1.14-1.59 |
| NSAIDs | **1.69** | 1.14-1.59 | **1.69** | 1.62-1.59 |

^a.^ Conditional logistic regression adjusted for important potential time-varying discordant exposure to medications potentially associated with heart failure in Table 3 plus co-morbidities including hypertension, ischemic heart disease, cerebrovascular disease, chronic kidney disease

^b^ Conditional logistic regression adjusted for important potential time-varying discordant exposure to medications potentially associated with heart failure in Table 3 plus surrogate measures of glycemic control including percentages of number of outpatients visits ≥2 and percentages of number of A1c test ≥1

**Bold** values indicate statistical significance.

Abbreviations: C.I., confidence interval; OR, odds ratio.

**Supplementary Table 3.** Proportion of patients with prescriptions of co-morbidities and complications, concomitant medications, and resource utilization during 1-30 days and 91-120 days before death (N = 55,458)

|  | Case period | Control period |
| --- | --- | --- |
| Co-morbidities and complications (%), con-concomitant medication (%), | (1-30 days before index day) | (91-120 days before index day) |
| *Concomitant medications (%)* |  |  |
| Metformin | 10.32 | 8.47 |
| Sulfonylurea | 8.27 | 6.73 |
| Alpha-glucosidase inhibitors | 1.78 | 1.36 |
| Pioglitazone | 0.46 | 0.47 |
| Glinides | 3.49 | 2.12 |
| DPP4 inhibitors | 1.74 | 1.44 |
| Sitagliptin | 1.41 | 1.21 |
| Saxagliptin | 0.17 | 0.12 |
| Vildagliptin | 0.10 | 0.07 |
| Insulin | 56.07 | 10.01 |
| Aspirin | 26.71 | 17.97 |
| Clopidogrel | 8.58 | 4.86 |
| Cilostazol | 2.45 | 1.55 |
| Warfarin | 3.47 | 2.70 |
| Angiotensin converting enzyme inhibitors | 10.27 | 7.61 |
| Angiotensin receptor blockers | 2.68 | 2.91 |
| Alpha-blockers | 4.26 | 4.14 |
| Beta-blockers | 24.87 | 18.34 |
| Calcium channel blockers | 41.99 | 31.40 |
| Diuretics | 68.86 | 34.83 |
| Other anti-hypertensive agents | 2.73 | 1.53 |
| Nitrates | 18.46 | 11.36 |
| Statins | 5.90 | 5.78 |
| Fibrates | 1.12 | 1.17 |
| Digitalis glycoside | 13.27 | 6.28 |
| Antiarrhythmics class I and III | 23.92 | 6.80 |
| Inhaled beta-agonists | 49.19 | 17.00 |
| Inhaled anticholinergics | 28.59 | 9.07 |
| Inhaled corticosteroids | 3.31 | 1.19 |
| Aminophylline | 22.62 | 16.57 |
| Oral corticosteroids | 27.79 | 19.40 |
| Systemic antibiotics | 84.17 | 37.21 |
| Non-steroid antiinflammatory drugs | 36.07 | 27.15 |
| Antidepressants | 8.82 | 7.75 |
| *Comorbidity and complications (%)* |  |  |
| Atrial fibrillation | 4.31 | 3.09 |
| Cancer | 30.51 | 25.06 |
| Chronic kidney disease | 9.38 | 6.93 |
| Chronic liver disease | 11.86 | 9.46 |
| Chronic lung disease | 20.53 | 16.98 |
| Depression | 1.86 | 2.45 |
| Hypertension | 24.85 | 28.32 |
| Intracerebral hemorrhage | 4.45 | 1.65 |
| Ischemic heart disease | 10.01 | 8.58 |
| Ischemic stroke | 7.33 | 6.29 |
| Myocardial infarction | 3.49 | 1.05 |
| Peripheral vascular disease | 0.50 | 0.56 |
| Diabetic neuropathy | 1.18 | 1.80 |
| Diabetic retinopathy | 0.22 | 0.29 |
| Cerebrovascular disease | 16.85 | 13.90 |
| *Resource utilization* |  |  |
| Mean number of outpatient visits | 2.65 | 3.18 |
| Mean number of A1c test | 0.20 | 0.10 |
| Number of outpatient visits ≥2 (%) | 57.20 | 65.29 |
| Number of A1c test ≥1 | 18.85 | 9.21 |

**Supplementary Table 4.** Risk of death associated with current use of study drugs (N = 55,458)

|  | Number of patients exposed to the medication | | | Crude odds ratio | | Adjusted odds ratio* | |
| --- | --- | --- | --- | --- | --- | --- | --- |
|  | During case  (1-30) period or control period (91-120) (%) | During case  (1-30) period but not control period (91-120) (%) | During control period (91-120) but not case period (1-30) (%) | Point estimate | 95% C.I. | Point  estimate | 95 % C.I |
| Cilostazol | 3.01 | 1.45 | 0.56 | **3.90** | 3.51-4.33 |  |  |
| NSAIDs | 48.21 | 21.05 | 12.14 | **2.24** | 2.19-2.30 |  |  |

*Conditional logistic regression adjusted for important potential time-varying confounding variables including pioglitazone, metformin, sitagliptin, aspirin,

angiotensin converting enzyme inhibitors, beta-blockers, calcium channel blockers, diuretics, nitrates, digitalis glycoside, inhaled beta-agonists, inhaled

anticholinergics, aminophylline, oral corticosteroids, and systemic antibiotics

**Bold** values indicate statistical significance, OR: odds ratio, 95% C.I.: confidence interval

**Supplementary Table 5.** Adjusted odds ratio

|  | A: Model 1 | | A: Model 2 | | A: Model 3 | | A: Model 4 | | A: Model 5 | | B | |
| --- | --- | --- | --- | --- | --- | --- | --- | --- | --- | --- | --- | --- |
|  | Point  estimate | 95 % C.I | Point  estimate | 95 % C.I | Point  estimate | 95 % C.I | Point  estimate | 95 % C.I | Point  estimate | 95 % C.I | Point  estimate | 95 % C.I |
| Cilostazol | 1.69 | 1.43-1.98 | 1.59 | 1.36-1.87 | 1.31 | 1.10-1.56 | 1.22 | 1.01-1.46 | 1.12 | 0.93-1.35 | 2.36 | 2.06-2.70 |
| NSAIDs | 1.25 | 1.20-1.30 | 1.22 | 1.17-1.27 | 1.10 | 1.06-1.15 | 1.05 | 1.01-1.10 | 0.86 | 0.82-0.90 | 1.67 | 1.62-1.72 |

A.

Model 1: Insulin

Model 2: Model 1 + aspirin

Model 3: Model 2 + beta-blockers, calcium channel blockers, diuretics, nitrates, digitalis glycoside, anti-arrhythmics Class I/III

Model 4: Model 3 + inhaled beta-agonists, inhaled anticholinergics, aminophylline, oral corticosteroids

Model 5: Model 4 + systemic antibiotics

B. atrial fibrillation, cancer, chronic kidney, liver, lung, hypertension, ischemic heart disease, cerebrovascular disease
